# Progressive obtundation in a young woman with bilateral corpus striatum infarction: a case report

**DOI:** 10.1186/1752-1947-5-324

**Published:** 2011-07-25

**Authors:** Osama SM Amin, Sa'ad Seud Shwani, Hero M Zangana, Nawa A Ameen

**Affiliations:** 1 Department of Neurology, Sulaimaniya General Teaching Hospital, Sulaimaniya City, Iraq; 2 Department of Medicine, Sulaimaniya General Teaching Hospital, Sulaimaniya City, Iraq

## Abstract

**Background:**

Bilateral ischemic infarction involving the corpus striatum is a rare event which usually results from global cerebral hypoxia, intoxications, and drug abuse.

**Case presentation:**

We report a 28 year old Caucasian woman who presented with progressive obtundation and later development of severe expressive dysphasia and Parkinsonism after sustaining ischemic stroke of both corpora striata. Hemorrhagic transformation developed on day four of admission.

**Conclusion:**

This is a rare case of bilateral basal ganglia infarction with hemorrhagic transformation in a young patient. Our patient's work up did not reveal any cause behind this stroke; however, advanced investigations (such as genetic testing and conventional angiography) were not done. The damage resulted in motor dysphasia and Parkinsonism. Neither dystonia nor other involuntary movements developed, and cognitive function was not assessed because of the language disorder.

## Background

The human basal ganglia, which have a complex anatomy and physiology, are supplied by several blood vessels on either side. Bilateral ischemic infarction involving the corpus striatum is a rare event which usually results from global cerebral hypoxia, intoxications, and drug abuse.

## Case report

**Figure 1:**
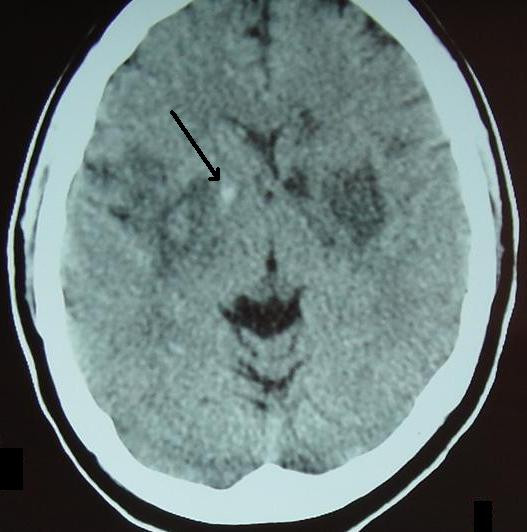
**Non contrast brain CT scan of our patient at the time of admission**. Note the bilateral hypodensities, which fit the area of the lenticular nucleus (putamen and globus pallidus) on both sides. There is also small hyperdensity at the right globus pallidus (black arrow). This prompted the physician to suspect an infectious process instead of a vascular one. Our patient had bilateral infarction of the lenticular nucleus with early hemorrhagic transformation inside the right one.

**Figure 2:**
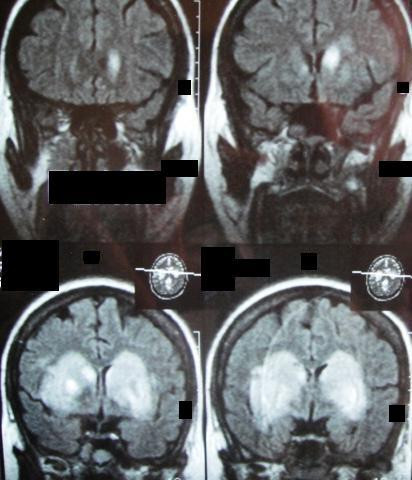
**Coronal T2 FLAIR (fluid attenuation inversion recovery) brain MRI film of our patient on the fifth day of hospital stay**. Note the bilateral heterogeneous hyper intensities at the right and left corpus striatum. These areas (which were not suppressed on this film) represent ischemic infarction with hemorrhagic transformation.

A 28 year old Caucasian woman was brought to our emergency department with a five hour history of progressive impairment in consciousness and slurred speech. Her past history was unremarkable, and she neither smoked nor drank alcohol. Her older brother said that she took no medications and she did not use illicit drugs as far as he knew. No history of head trauma was obtained. At the time of admission, her blood pressure was 140/70 mmHg with a pulse rate of 90 beats/minute, respiratory rate of 12 cycles/minute, and a temperature 37.1°C. Our patient was stuporous and there were no lateralizing signs or neck stiffness. Both planter reflexes were down. Our patient underwent a battery of investigations with the following results: hemoglobin 13.6 g/L; total white cell count 9100/mL^3^; platelets 270,000/mL^3^; mean corpuscular volume 84fL; mean corpuscular hemoglobin concentration 33 g/dL; erythrocyte sedimentation rate 19 mm/hour; blood urea 35 mg/dL; serum creatinine 0.9 mg/dL; serum sodium 139 mEq/L; serum potassium 4.1 mEq/L; serum calcium 8.9 mg/dL; serum total bilirubin 0.8 mg/dL; aspartate transaminase 21 u/L; alanine transaminase 19 u/L; alkaline phosphatase 190 u/L; serum total protein 7.3 g/dL; serum albumin 4.4 g/dL; thyroid stimulating hormone 2.9 u/L; serum total triiodothyronine 1.3 nmol/L; serum total thyroxin 89 nmol/L; serum total cholesterol 177 mg/dL; serum triglyceride 100 mg/dL; low density lipoprotein cholesterol 128 mg/dL; very low density lipoprotein cholesterol 20 mg/dl; high density lipoprotein cholesterol 38 mg/dl; prothrombin time 12 seconds; activated partial thromboplastin time 31 seconds; a serum Venereal Disease Research Laboratory test was negative; and general urine examination and microscopy were unremarkable. Blood and urinary screening for cocaine, opioids and amphetamines was negative. A 12 lead electrocardiogram (ECG) was normal. A non contrast brain computed tomography (CT) scan showed bilateral hypodensities in her corpus striatum (Figure 1). In addition, there was a small hyper dense area at the anterior part of her right globus pallidus. The physician suspected encephalitis, and managed our patient accordingly. He ordered serology for toxoplasma and human immunodeficiency virus, and a lumber puncture was done: all of these tests turned out to be negative. At day four of admission, our patient became comatose, and our neurology department was consulted. The Glasgow coma scale was 3/15, no neck stiffness was detected, and both planter reflexes were up. On day five, a brain magnetic resonance imaging (MRI) scan with gadolinium revealed hemorrhagic infarctions involving both basal ganglia (Figure 2). Brain magnetic resonance angiography and magnetic resonance venography (MRV) were normal. Serum anti nuclear and rheumatoid factors as well as anti phospholipid antibodies were negative. Transthoracic and transesophageal echocardiographic examinations were normal, as was the carotid Doppler study.

Our patient was managed as ischemic stroke with secondary hemorrhagic transformation. Anti hypertensives and a statin were prescribed. Anti platelets and anticoagulation were not given. Gradually over a period of three weeks, our patient's consciousness improved to a degree of mild drowsiness. As for her language assessment, comprehension was intact but there was no speech output; she uttered few sounds, however, but no comprehensible words. She had generalized rigidity and hypokinesia. No abnormal movements were found and dystonic posturing was absent.

## Discussion

The corpus striatum (which forms the bulk of the basal ganglia) is composed of the neostriatum (made up of putamen, caudate nucleus, and nucleus accumbens) and the paleostriatum (with its internal and external segments of globus pallidus as well as the ventral pallidum) [1] In their study, Feekes and Cassell [2] found that the human corpus striatum's blood supply comes principally from the medial and lateral lenticulostriate branches of M1 and M2 segments of the middle cerebral artery and from the recurrent artery of Huebner (which stems from the A2 segment of the anterior cerebral artery). The anterior choroidal and anterior communicating arteries have a minor contribution. The middle cerebral artery also gives off direct small perforators to the striatum, but these blood vessels contribute very little to the overall blood supply [3].

Therefore, acute and extensive ischemic damage of both corpora striata mainly requires occlusion of deep perforating lenticulostriate branches of both middle cerebral arteries and the arteries of Huebner.

Theoretically, multiple emboli to these blood vessels can produce bilateral basal ganglia infarction. Russmann *et al*. [4] found that eight out of their 13 patients with extensive lenticular infarction had an embolic cause (artery to artery in four patients, cardioembolism in three patients and one undetermined source). The unremarkable ECG as well as echocardiographic and carotid Doppler studies ruled out an embolic source.

Stam [5] suggested that cerebral venous sinus thrombosis should be suspected in patients with brain CT evidence of hemorrhagic infarctions, especially if these infarctions were multiple and did not follow a specific arterial territory (as in our patient). The patient's brain MRV was normal, however. We reviewed the brain MRI and MRV with two radiologists; they disagreed with cerebral venous sinus thrombosis as an etiology. The sensitivity of combined brain MRI/MRV in the diagnosis of cerebral venous sinus thrombosis is high [6,7]. In addition, systemic lupus erythematosus and anti phospholipid syndrome were on the differential diagnosis list. The negative clinical and laboratory work up cancelled out these options.

Bilateral infarction of the corpus striatum is a well documented event as an aftermath of pan cerebral hypoperfusion [8], intoxications and poisoning (such as cyanide [9] and carbon monoxide [10]), illicit drug use (for example cocaine) [11], head trauma [12], and supratentorial neurosurgical procedures [13]. None of these factors was operative in our patient.

Hawker and Lange [8] found that pancerebral hypoxia and ischemia are more likely to damage the globus pallidus; the putamen ranks second. The overall clinical picture also varies, ranging from akinetic rigid syndrome to pure dystonia. According to Grandas *et al*. [9], cyanide poisoning destroys the putamen and external segments of the globus pallidus; this combination results in severe Parkinsonism and progressive dystonia. Approximately 13% of patients with carbon monoxide poisoning develop delayed motor disorders, according to Quinn *et al*. [10]; a variable combination of Parkinsonism, dystonia, chorea, and myoclonus ensue.

Renard *et al*. [11] concluded that bilateral hemorrhagic infarction of basal ganglia usually occurs when cocaine is co-administered with heroin, rather than after cocaine abuse alone. Ishihara *et al*. [12] reported a case of bilateral basal ganglia infarction in an 11-month-old child who sustained a mild head trauma to his forehead. Von Eckardstein and his neurosurgical team [13] performed an operation on a 68 year old woman and removed a right parietal parasagittal dural tumor with reconstruction of the right wall of the superior sagittal sinus; postoperatively, the patient remained unresponsive and brain imaging revealed bilateral basal ganglia infarction.

Due to the lack of expertise in our radiology department, conventional cerebral angiography was not done. The negative evaluation of the cause behind this patient's stroke would categorize our patient as having a "stroke of undetermined etiology," according to the TOAST classification [14]. However, as our work up lacks several advanced investigations (such as genetic testing and cerebral angiography) this categorization cannot be done [15].

Isolated and discrete lesions involving various structures of corpus striatum usually result in specific clinical features. For instance, damage to the anterioventral caudate can cause contra lateral choreoathetosis [16]. It should be noted that the ischemic infarction rarely confines itself strictly to the corpus striatum; it usually involves nearby structures, such as thalamus, hypothalamus, and internal capsule and other white matter projection fibers. Therefore, the precise correlation between bilateral lesions of corpus striatum and the resulting cognitive, language, and motor dysfunction is usually blurred.

Our patient's presentation of progressive obtundation can be explained by the bilateral deep hemispheric dysfunction. During her recovery, our patient demonstrated severe expressive dysphasia (rather than abulia). Mega and Alexander [17] suggested that this form of subcortical dysphasia results from damage to the frontocaudate functional system and the connecting deep white matter fibers. According to Bhatia and Marsden [18], her Parkinsonism can be ascribed to bilateral lesions in the putamen and/or globus pallidus. Cognitive and behavioral abnormalities are very common in basal ganglia lesions, especially bilateral ones [19]. Our patient's severe language dysfunction rendered cognitive function assessment virtually impossible.

Our patient was discharged five weeks after admission. She came back for a scheduled follow up visit after one month. She still had severe expressive dysphasia and moderate hypokinesia and rigidity. After careful questioning, the family denied any form of involuntary movements or dystonia. Giroud *et al*. [20] found that dystonia was the commonest consequence of lenticular damage (whether acute or chronic). On the other hand, Russmann *et al*. [4] concluded that dystonia was a rare sequela to lenticular (putamen and globus pallidus) lesions, a finding that is consistent with ours.

## Conclusion

This is a rare case of bilateral basal ganglia infarction with hemorrhagic transformation in a patient. The patient's work up did not reveal any cause behind this stroke; however, advanced investigations (such as genetic testing and conventional angiography) were not done. The damage resulted in motor dysphasia and Parkinsonism. Neither dystonia nor other involuntary movements developed, and cognitive function was not assessed because of the language disorder.

## Consent

Written informed consent was obtained from the patient for publication of this case report and any accompanying images. A copy of the written consent is available for review by the Editor-in-Chief of this journal.

## Competing interests

The authors declare that they have no competing interests.

## Authors' contributions

Clinical work up was made by OSMA and SSS. SSS took the photos of the brain imaging. The literature search was done by OSMA. HMZ and NAA undertook patient follow up. OSMA wrote the manuscript; all authors read and approved the final manuscript.

## Authors' information

OSMA is a board certified neurologist and a Fellow of the American College of Physicians. SSS is a registrar in clinical adult neurology. HMZ is a neurology trainee. NAA is an intern at the department of internal medicine and neurology.
